# Molecular evolution, characterization, and expression analysis of SnRK2 gene family in Pak-choi (*Brassica rapa* ssp. *chinensis*)

**DOI:** 10.3389/fpls.2015.00879

**Published:** 2015-10-21

**Authors:** Zhinan Huang, Jun Tang, Weike Duan, Zhen Wang, Xiaoming Song, Xilin Hou

**Affiliations:** ^1^State Key Laboratory of Crop Genetics and Germplasm Enhancement, Key Laboratory of Biology and Germplasm Enhancement of Horticultural Crops in East China, College of Horticulture, Nanjing Agricultural UniversityNanjing, China; ^2^Institute of Horticulture, Jiangsu Academy of Agricultural ScienceNanjing, China

**Keywords:** evolution pattern, Pak-choi, SnRK2, cold stress, ABA, expression profile

## Abstract

The sucrose non-fermenting 1-related protein kinase 2 (SnRK2) family members are plant-specific serine/threonine kinases that are involved in the plant response to abiotic stress and abscisic acid (ABA)-dependent plant development. Further understanding of the evolutionary history and expression characteristics of these genes will help to elucidate the mechanisms of the stress tolerance in Pak-choi, an important green leafy vegetable in China. Thus, we investigated the evolutionary patterns, footprints and conservation of *SnRK2* genes in selected plants and later cloned and analyzed *SnRK2* genes in Pak-choi. We found that this gene family was preferentially retained in Brassicas after the *Brassica-Arabidopsis thaliana* split. Next, we cloned and sequenced 13 SnRK2 from both cDNA and DNA libraries of stress-induced Pak-choi, which were under conditions of ABA, salinity, cold, heat, and osmotic treatments. Most of the *BcSnRK2s* have eight exons and could be divided into three groups. The subcellular localization predictions suggested that the putative BcSnRK2 proteins were enriched in the nucleus. The results of an analysis of the expression patterns of the *BcSnRK2* genes showed that *BcSnRK2* group III genes were robustly induced by ABA treatments. Most of the *BcSnRK2* genes were activated by low temperature, and the *BcSnRK2.6* genes responded to both ABA and low temperature. In fact, most of the *BcSnRK2* genes showed positive or negative regulation under ABA and low temperature treatments, suggesting that they may be global regulators that function at the intersection of multiple signaling pathways to play important roles in Pak-choi stress responses.

## Introduction

In nature, plants are frequently exposed to harmful environmental conditions, such as low or high temperature, high salinity, and drought. To cope with these stresses by surviving and completing their life cycles, plants have evolved complex physiological and genetic mechanisms (Bohnert et al., 2006; Fujita et al., 2006). Protein phosphorylation plays a key role in plant growth and development by regulating such processes as cell division, metabolism, and intracellular signal transduction. The protein kinases involved in stress signal transduction in plants are common to all eukaryotic organisms and include mitogen-activated protein kinases (MAPKs), glycogen synthase kinase 3 (GSK3), S6 kinase (S6K). Plant specific kinases also play a role, for example, calcium-dependent protein kinases (CDPKs) and most of sucrose non-fermenting 1-related kinases (SnRKs; Cho et al., 2009; Kulik et al., 2011).

SnRK2 is a plant-specific Ser/Thr protein kinase family. All of the members have a conserved N-terminal catalytic domain similar to SNF1/AMP kinases and a short C- terminal regulatory domain that is not highly conserved. Prior to 2000, there were only a small number of studies indicating that ABA and abiotic stresses induce the expression of some *SnRK2* genes (Anderberg and Walker-Simmons, 1992; Holappa and Walker-Simmons, 1995). In 2000, SnRK2s began to be recognized as enzymes involved in abiotic stress signal transduction in plants (Li et al., 2000). By 2003, 10 *SnRK2* genes had been identified and were renamed *SnRK2.1* through *SnRK2.10* (Hrabak et al., 2003). In 2009, independently, two laboratories obtained a triple *SnRK2.2/3/6* mutant. The *SnRK2.2/2.3/2.6* triple-mutant plants are nearly completely insensitive to ABA, which was used to establish the role of ABA dependent SnRK2s in the plant response to water deficit, seed maturation, and germination. These reports indicate that *SnRK2.2/3/6* function as primary positive regulators, and suggest that ABA signaling is controlled by the dual modulation of *SnRK2.2/3/6* and group-A *PP2Cs* (Fujii and Zhu, 2009; Fujii et al., 2009; Nakashima et al., 2009). In 2013, SnRK2 protein kinase substrates were identified by Zhu's laboratory (Wang et al., 2013), including proteins involved in flowering time regulation, RNA and DNA binding, miRNA and epigenetic regulation, signal transduction, chloroplast function, and many other cellular processes. This work significantly contributed to the understanding of the role of SnRK2 protein kinases and the downstream effectors of ABA action.

Currently, with the identification of the primary components, including SnRK2, ABFs, AFPs, group-A PP2C, and PYRs in algal (Wang et al., 2015), the function of the ABA signaling pathway could traced back to before the divergence of land plants. Angiosperm genome evolution is characterized by polyploidization through whole-genome duplication (WGD) followed by diploidization, which is typically accompanied by considerable homoeologous gene loss (Stebbins, 1950). For example, the genome of *Arabidopsis thaliana* has experienced a paleohexaploidy (γ) duplication shared with most dicots and two subsequent genome duplications (α and β) since its divergence from *Carica papaya* along with a rapid DNA sequence divergence and extensive gene loss (fractionation; Bowers et al., 2003). *C. papaya, Populus trichocarpa*, and *Vitis vinifera* did not undergo α and β duplications, and *Amborella trichopoda* did not undergo γ duplication. In addition, lycophyte, physcomitrella, and chlorophyta did not experience any WGD event. After the WGD events, several gene copies with products that participate in macromolecular complexes or in transcriptional or signaling networks were preferentially retained to avoid network instability caused by the loss of one member (Birchler and Veitia, 2007). In *A. thaliana*, sorghum and rice, 10 *SnRK2* genes have been well-annotated (Kolukisaoglu et al., 2004; Kulik et al., 2011). Based on former reports, *SnRK2* genes can be divided into three groups, and group III is the most ancient group and is involved in the ABA signal pathway (Hauser et al., 2011). However, the retention, conservation and detailed mechanisms of SnRK2 gene family evolution, especially for group III, in the angiosperms that experienced different WGD events has not been thoroughly elucidated.

“U”s triangle Brassicas crops, which are major contributors to the human diet and were among the earliest cultigens, were included in Brassicaceae with the model plant *A. thaliana*. The triangle consisted of the three diploid species, *Brassica rapa* (A genome, 2*n* = 20), *Brassica nigra* (B genome, 2*n* = 16), and *Brassica oleracea* (C genome, 2*n* = 18), having formed the amphidiploid species *Brassica juncea* (A and B genomes, 2*n* = 36) by hybridization, *Brassica napus* (A and C genomes, 2*n* = 38), and *Brassica carinata* (B and C genomes, 2*n* = 34). These species shared a complex history with *A. thaliana* and experienced an additional whole-genome triplication (WGT) event 13–17 million years ago (MYA) (Wang et al., 2011; Cheng et al., 2013). To date, *B. rapa* and *B. oleracea* were recently sequenced and assembled (Wang et al., 2011; Liu et al., 2014), providing good resource material to study the evolutionary patterns of *SnRK2* genes. In addition, *B. rapa*, AA genome, contains Chinese cabbage (*B. rapa* ssp. *pekinensis*) and non-heading Chinese cabbage (*B. rapa* ssp. *Chinensis*; NHCC; Pak-choi). Both of these species are economically significant vegetable crops in Asia, and Pak-choi is one of the most important vegetables in Southeast China due to its high yield and good quality. During the planting process, the environmental stresses that always appear in Southeast China, which include heat, cold, water logging, and salinity, affect the production of Pak-choi. This process is important to increase the stress resistance of Pak-choi. However, little is known regarding the molecular characteristics of *SnRK2* genes in Pak-choi.

To elucidate the evolutionary pattern, retention and conservation of SnRK2 genes and to characterize SnRK2 gene expression in Pak-choi further, we performed the following: (i) identified the divergence and footprint of *SnRK2* genes during γ, α, and β WGD events through 5 eudicots, 1 basal angiosperm, 1 lycophyte, 1 physcomitrella, and 1 chlorophyta; (ii) compared the conservation of *SnRK2* genes in Brassicas AA and CC genomes; (iii) cloned and characterized 13 putative *BcSnRK2* genes from stress-inducible Pak-choi cDNA and DNA libraries; (iv) investigated the molecular features and gene structure of *BcSnRK2* gene family members; (v) analyzed *BcSnRK2* gene family expression profiles under ABA and low temperature treatment; (vi) identified genes that co-responded to ABA and cold stress, and investigated the co-regulation networks and the divergence of the functions of *BcSnRK2* genes. This study is the first report on *SnRK2* genes in Pak-choi and extends our understanding of the roles of the *SnRK2* gene family in cold stress responses and ABA signaling. The results provide support for the breeding of Pak-choi.

## Materials and methods

### Database search

The coding sequences of 10 *A. thaliana* SnRK2 genes that constituted the set of SnRK2 genes used in this study were retrieved from previous reports (Hrabak et al., 2003; Fujii et al., 2007). The genome sequences of *B. rapa, B. oleracea*, and *A. trichopoda* were downloaded from the BRAD (http://brassicadb.org/brad/; Wang et al., 2011), and Amborella Genome Database (http://www.amborella.org/; Albert et al., 2013). The gene information of *V. vinifera, C. papaya, P. trichocarpa, Physcomitrella patens, Chlamydomonas reinhardtii*, and *Selaginella moellendorffii* were downloaded from Phytozome v9.1 (http://phytozome.jgi.doe.gov/pz/portal.html; Goodstein et al., 2012). Next, 10 *AthSnRK2* genes were used as seed sequences to identify SnRK2 homologous genes using BLASTn searches (*E*-value threshold 1e-10) of *B. rapa* and seven other plant species, including 3 eudicots (*C. papaya, P. trichocarpa*, and *V. vinifera*), 1 basal angiosperm (*A. trichopoda*), 1 lycophyte (*S. moellendorffii*), 1 physcomitrella (*P. patens*), and 1 chlorophyta (*C. reinhardtii*).

### Plant materials, growth conditions, and stress treatments

Pak-choi (*B. rapa* ssp. *chinensis cv. suzhouqing*) was used for all experiments. Seedlings were soaked in distilled water for 0.5 h and later germinated in plastic Petri dishes containing filter paper saturated with distilled water in darkness at 22°C for 2 days. Seedlings were subsequently transferred to 4 L hydroponic containers containing continuously aerated 1/2 Murashige and Skoog (MS) liquid solution (pH 5.8, without agar and sugar). The 1/2 MS liquid solution was changed once every 3 days. Three-week-old seedlings were transferred to new 1/2 MS liquid solution (pH 5.8, without agar and sugar) for multiple stress treatments under a continuous time course (0, 1, 6, 12, 24, and 48 h). For ABA, salt and osmotic treatments, seedlings were exposed to 1/2 MS solution (pH 5.8) containing 100-μM ABA, 200-mM NaCl, and 15% (w/v) polyethylene glycol (PEG), respectively. For cold and heat treatments, seedlings were exposed to the 4–38°C conditions in 1/2 MS solution (pH 5.8), respectively. All seedlings were placed under the same growth conditions, except for the different treatment factors, and exposed to 1/2 MS solution at 22°C as were controls. The seedlings were harvested under a continuous time course (0, 1, 6, 12, 24, and 48 h) in three biological replicates for RNA preparation (Tang et al., 2013).

Total RNA and DNA were isolated from 100 mg of frozen Pak-choi tissues under multiple abiotic stress conditions using an RNA kit (RNAsimply total RNA Kit, Tiangen, Beijing, China) and an DNA kit (MiniBEST Plant Genomic DNA Extraction Kit, TaKaRa, Daliang, China), respectively, according to the manufacturer's instructions. The quality and quantity of every RNA sample was assessed by agarose gel electrophoresis.

### Cloning and identification of the BcSnRK2s members in pak-choi

To clone *BcSnRK2* genes, we designed gene-specific primers (Supplementary Table 1) based on CDS sequences of Chinese cabbage SnRK2 orthologs to amplify the full-length cDNA and DNA sequences of BcSnRK2 in the stress-induced Pak-choi cDNA and DNA libraries.

After obtaining all of the SnRK2 genomic and ORF sequences of Pak-choi, we verified these sequences using SMART (http://smart.embl-heidelberg.de/; Ludwig-Müller, 2011) and Pfam (http://pfam.janelia.org/).

### Gene feature analysis, conserved motif analysis, and subcellular localization prediction

We research the gene structure using Gene Structure Display Server (GSDS; http://gsds.cbi.pku.edu.cn/) and the feathers of genes using EBI-Tools (http://www.ebi.ac.uk/Tools/emboss/). To identify the C-terminal conserved motifs of the SnRK2s, we used MEME (http://meme.sdsc.edu/meme/meme.html; Bailey et al., 2009) with the motif length set at 10–100 and motif maximum number set at 10. Protein subcellular localization was predicted using WoLF PSORT (http://wolfpsort.org/; Horton et al., 2007).

### Phylogenetic and structural conserved analysis

The protein sequences of SnRK2s were aligned using the MUSCLE program (Edgar, 2004) with default parameters. Next, the Phylogenetic tree was plotted using MEGA5 (Tamura et al., 2011) software with the Maximum likelihood (ML) method and 1000 bootstrap replicates. We used ConSurf sever (http://consurf.tau.ac.il; Ashkenazy et al., 2010) to establish the presumed protein module on *AthSnRK2.6* (PDB ID: 3UJG).

### Subcellular localization

To investigate the subcellular localizations of BcSnRK2 genes, two expression vectors were constructed using a transient expression system in onion epidermal cells. The full-length coding sequences of BcSnRK2.6a were amplified using Gateway-specific primers cloned into an entry vector, and then subcloned into pEarleyGate101 using Gateway technology (Invitrogen, Carlsbad, CA). The yellow fluorescent marker protein (YFP) was fused to BcSnRK2.6a. Gold particles with a diameter of 1 μm coated with 35S BcSnRK2.6a-YFP was introduced into onion epidermal cells using particle bombardment (PDS-100/He particle delivery system; Bio-Rad, Hercules, CA). After incubation at 22°C for at least 12 h under darkness, fluorescence and bright-light images were observed using laser scanning confocal microscopy (Leica, TCS SP2, Wetzlar, Germany).

### Quantitative real-time PCR analysis

The cDNA was synthesized from the total RNA using the Prime Script RT reagent Kit (TaKaRa). The specific sequences of the primers used for real time PCR are listed in Supplementary Table 1. To evaluate primer specificity, we used the BLAST program against the Brassica Genome. The reactions were performed using MyiQ Single color Real-Time PCR Detection System (Bio-Rad, Hercules, CA). The PCR conditions were 94°C for 30 s, 40 cycles at 94°C for 10 s, and 58°C for 30 s, followed by a melting curve (61 cycles at 65°C for 10 s) to determine the specificity of the amplification. Relative fold expression changes were calculated using the comparative C_t_ value method.

### Pearson correlation analyses

Pearson correlation coefficients (PCCs) of transcript levels of *BcSnRK2s* pairs were calculated using a house Perl script based on transformed qRT-PCR data under ABA and low temperature treatments. Correlation was analyzed using the R package program according to the PCCs. Gene co-regulatory network were constructed using Cytoscape version 3.1 based on the PCCs of these gene pairs that exhibited a *p*-value significance level of 0.05 (Shannon et al., 2003).

## Results

### Evolutionary history of SnRK2 genes

**Figure 1:**
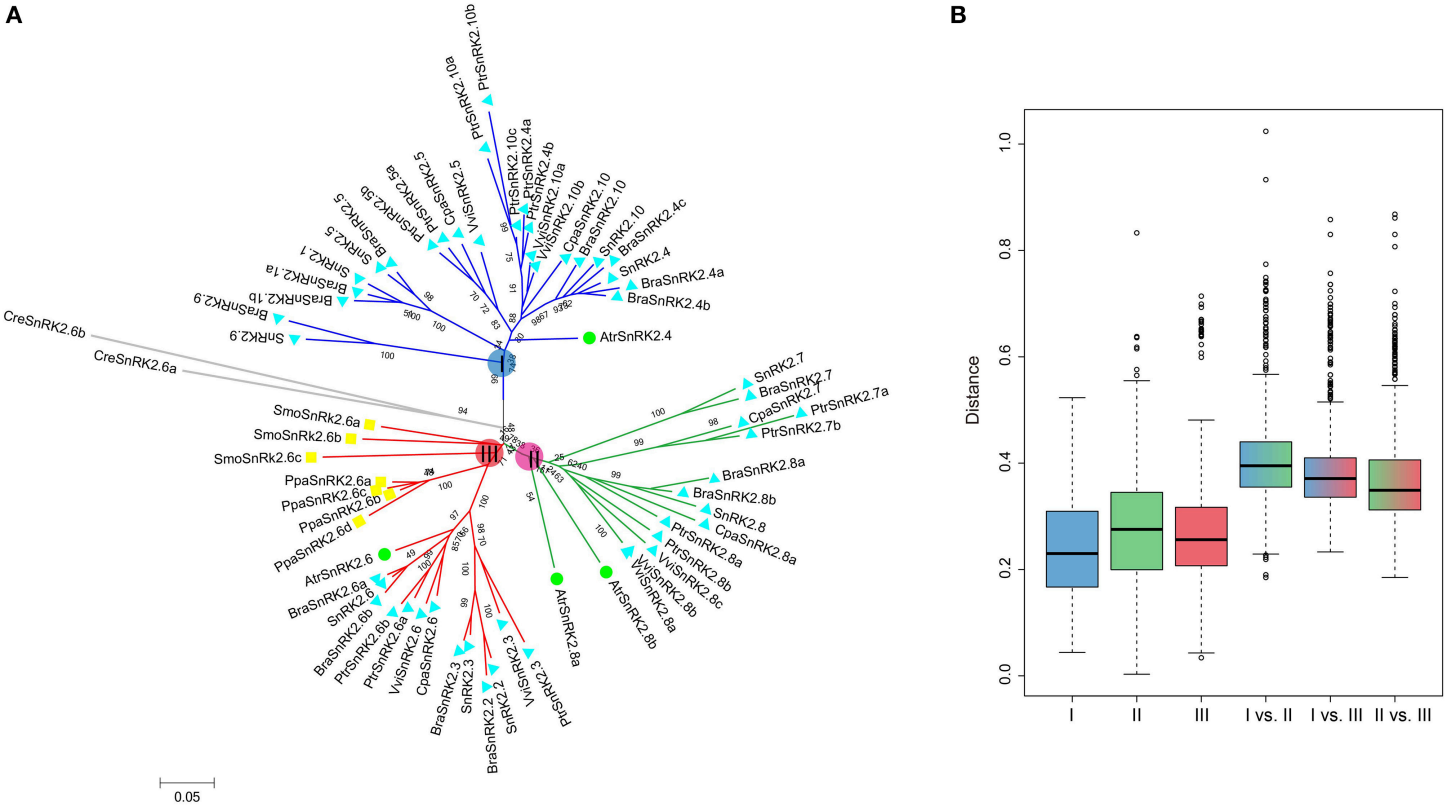
**Phylogenetic relationships among SnRK2 genes in nine plant species (A) and the genetic distance among different groups of SnRK2 genes (B)**. Group I, II, and III are shaded blue, green, and red, respectively. In **(A)**, the short species names are *C. reinhardtii* (Cre), *P. patens* (Ppa), *S. moellendorffii* (Smo), *A. trichopoda* (Atr), *V. vinifera* (Vvi), *P. trichocarpa* (Ptr), *C. papaya* (Cpa), *B. rapa* (Bra), and *A. thaliana* (Ath). The phylogenetic tree was constructed using maximum likelihood (ML) and bootstrap values calculated with 1000 replications using MEGA5 (Tamura et al., 2011). The SnRK2s of *A. trichopoda* were indicated by green balls, *S. moellendorffii* and *P. patens* were indicated by yellow squares, others were indicated by blue triangles.

All of the 10 *SnRK2* genes were identified in *A. thaliana* and clustered into groups I, II, and III (Fujii et al., 2007). To understand the evolutionary history of *SnRK2* genes in the plant kingdom, *SnRK2* homologous genes in *B. rapa, A. thaliana* and seven other plant species were analyzed. Overall, we identified 15 *BraSnRK2s*, 6 *CpaSnRK2s*, 14 *PtrSnRK2s*, 8 *VviSnRK2s*, 4 *AtrSnRK2s*, 4 *PpaSnRK2s*, 3 *SmoSnRK2s*, and 2 *CreSnRK2s* (Supplementary Tables 2, 3) and constructed an unrooted phylogenetic tree using these genes (Figure 1A). With the exception of *SmoSnRK2s* and *PpaSnRK2s* all of the genes in the tree clustered into group III, and the *SnRK2* genes in *C. reinhardtii, P. patens*, and *S. moellendorffii* were homologous to *SnRK2.6*, suggesting that the *SnRK2.6* and group III genes were the most ancient gene and group, respectively, among the *SnRK2* genes. Additionally, the genetic distances among these three groups were analyzed and shown in Figure 1B. Group II and group III were the closest relationship and group I and group II were the farthest relationship. Given previous findings that group III is the most ancient and group I is the most recent group (Umezawa et al., 2010), we inferred that both group I and group II formed from group III.

**Figure 2:**
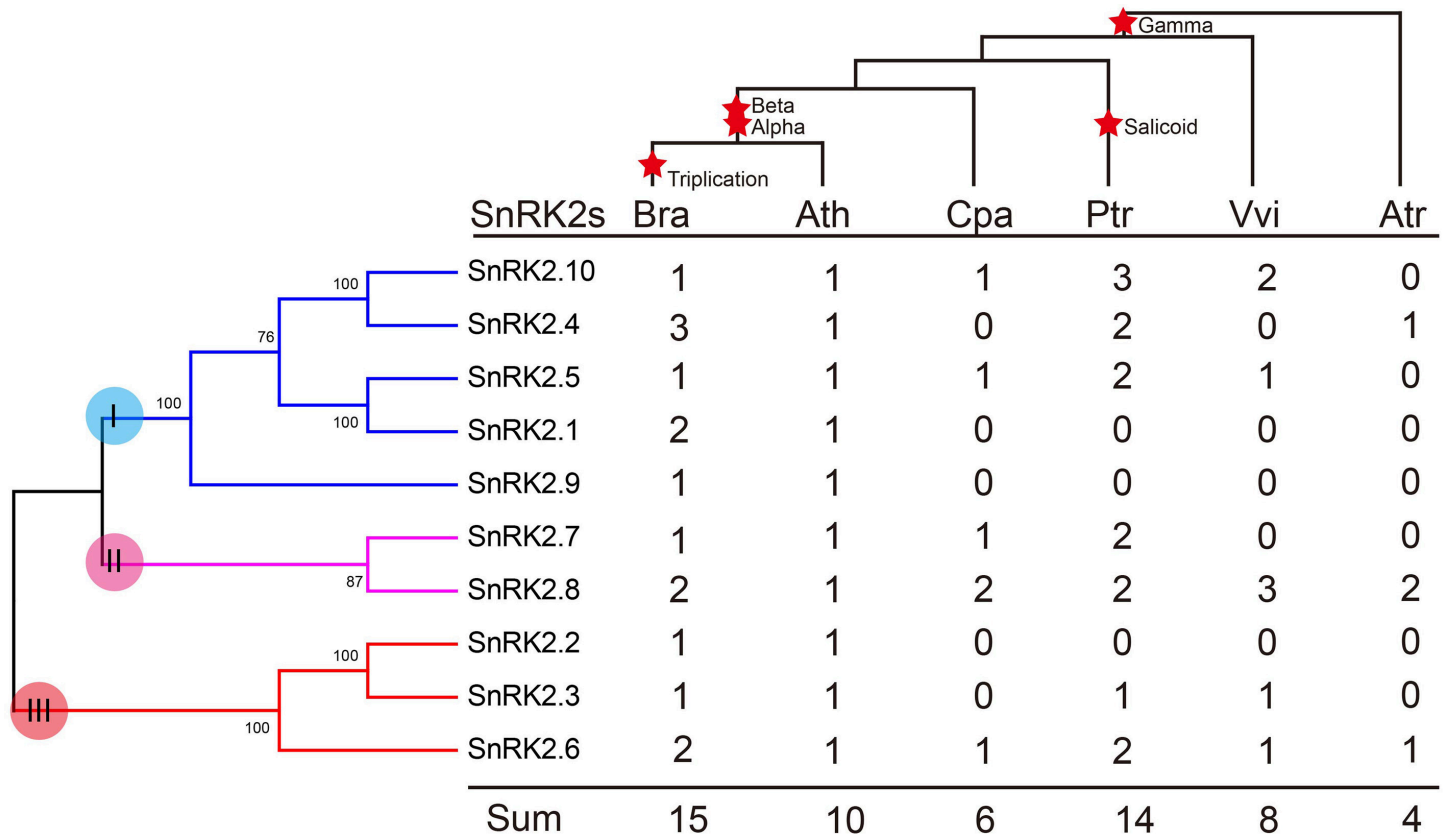
**Copy number variation in the SnRK2 family in plants**. The phylogenetic tree of SnRK2 genes is shown on the left, and the species tree is shown at the top. The α, β, γ, and salicoid duplications and the Brassica-specific triplication are indicated on the branches of the trees according to the Plant Genome Duplication Database. Numbers are copy numbers of each gene in *A. trichopoda* (Atr), *V. vinifera* (Vvi), *P. trichocarpa* (Ptr), *C. papaya* (Cpa), *B. rapa* (Bra), and *A. thaliana* (Ath).

We further investigated the footprint of the SnRK2 gene family in the selected angiosperms, *C. papaya, P. trichocarpa*, and *V. vinifera*, which did not undergo α and β duplications, and *A. trichopoda*, which did not undergo γ duplication. Phylogenetic trees in each species were constructed (Supplementary Figure 1). All trees could be divided into three groups, suggesting these groups originated prior to the γ event. Next, the copy numbers of the *SnRK2s* genes in each species were quantified (Figure 2). According to the results, *SnRK2.4, SnRK2.6, and SnRK2.8* appeared before the γ event; after the γ event, *SnRK2.3, SnRK2.5, SnRK2.7*, and *SnRK2.10* appeared. *SnRK2.1, SnRK2.2*, and *SnRK2.9* appeared after α and β duplications in *A. thaliana* and *B. rapa*. Furthermore, due to the salicoid duplication and *Brassica*-specific WGT events, there were more SnRK2 gene family members in *P. trichocarpa* and *B. rapa* than in other species (Tuskan et al., 2006; Wang et al., 2011). In general, during the course of evolution, group I appeared most recently and expanded most rapidly. Above all, we inferred a possible evolutionary footprint of the SnRK2 family (Supplementary Figure 2). Initially, the SnRK2 gene family had only *SnRK2.6*, which was included in group III; then three groups appeared before the γ event. After the divergence of Brassicales (α and β events), the size of the family increased to 10 due to the appearance of *SnRK2.1, SnRK2.2*, and *SnRK2.9*, and then it increased by half after triplication and fractionation. The expansion of group I played a major role in the expansion of the SnRK2 gene family.

### Conservation of the SnRK2 genes following whole genome triplication in brassicas

**Figure 3:**
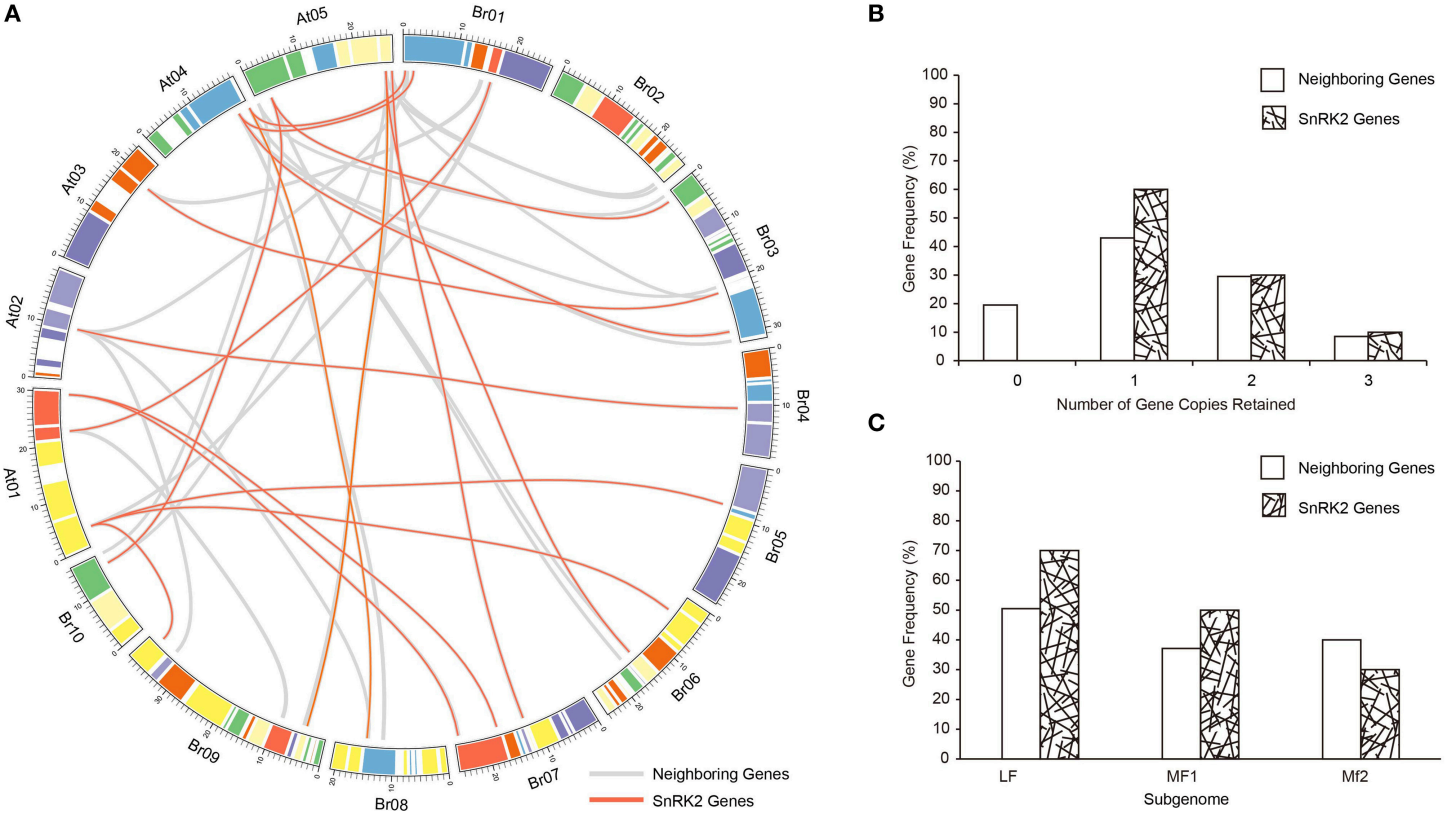
**Retention of SnRK2 genes and neighboring genes (10 flanking genes on either side of the clock gene) in *B. rapa.* (A)** Collinear correlations of SnRK2 genes and neighboring genes in the *A. thaliana* and *B. rapa* genomes. The *B. rapa* and *A. thaliana* chromosomes are colored according to the inferred ancestral chromosomes following an established convention. The lines of SnRK2 genes are red and neighboring genes are gray. The figure was created using Circos software. **(B)** Retention by number of homoeologous copies in the syntenic region. **(C)** Retention of homoeologs among the three subgenomes of *B. rapa*.

The whole genome of the Brassicas genome including *B. rapa* (AA) and *B. oleracea* (CC) have been well-studied, and three subgenomes have been established to distinguished the degree of fractionation (gene loss; Wang et al., 2011; Cheng et al., 2012). To evaluate the extent of fractionation of the *SnRK2* genes in Brassicas, the syntenic genes of *A. thaliana SnRK2* genes were also identified in *B. oleracea*, and all *BraSnRK2s* and *BolSnRK2s* were located in three subgenomes (Supplementary Table 4). Interestingly, all *SnRK2s* were retained in *B. rapa* and *B. oleracea* after the WGT. To further investigate the specific retention of *BraSnRK2* genes, the retention of 200 neighboring genes (10 on either side) flanking the 10 *A. thaliana SnRK2* genes (Supplementary Table 5) were compared in *B. rapa* genome and the syntenic relationship of all these genes between *B. rapa* and *A. thaliana* were displayed using the circos program (Figure 3A). Most (60%) *SnRK2* genes were retained in one copy, which is significantly greater than the retention of neighboring genes (Figure 3B). Almost similar numbers (30 and 31.5%) of *SnRK2* genes and neighboring genes were retained in two copies, while more (10%) of the *SnRK2* genes were retained in three copies than the neighboring genes (8.5%). Compared to the neighboring genes, significantly more *SnRK2* gene homologs were retained in the LF and MF1 subgenomes. The distribution of *SnRK2* genes in LF subgenome was greater than in other subgenomes, consistent with the whole genome level (Figure 3C). In addition, based on *K*_*s*_- values of all syntenic orthologs in *B. rapa* relative to *A. thaliana* (~0.42–0.45, ~14.5 million) and *SnRK2* genes in *B. rapa* relative to *A. thaliana* (~0.41), we speculated that the divergence of *BraSnRK2* genes were accompanied with a WGT event (Supplementary Table 6, Supplementary Figure 3). Overall, the *SnRK2* genes were preferentially retained, consistent with the gene balance hypothesis and demonstrating the significance of SnRK2 in plants.

### Isolation and identification of SnRK2 gene family members from pak-choi

**Figure 4:**
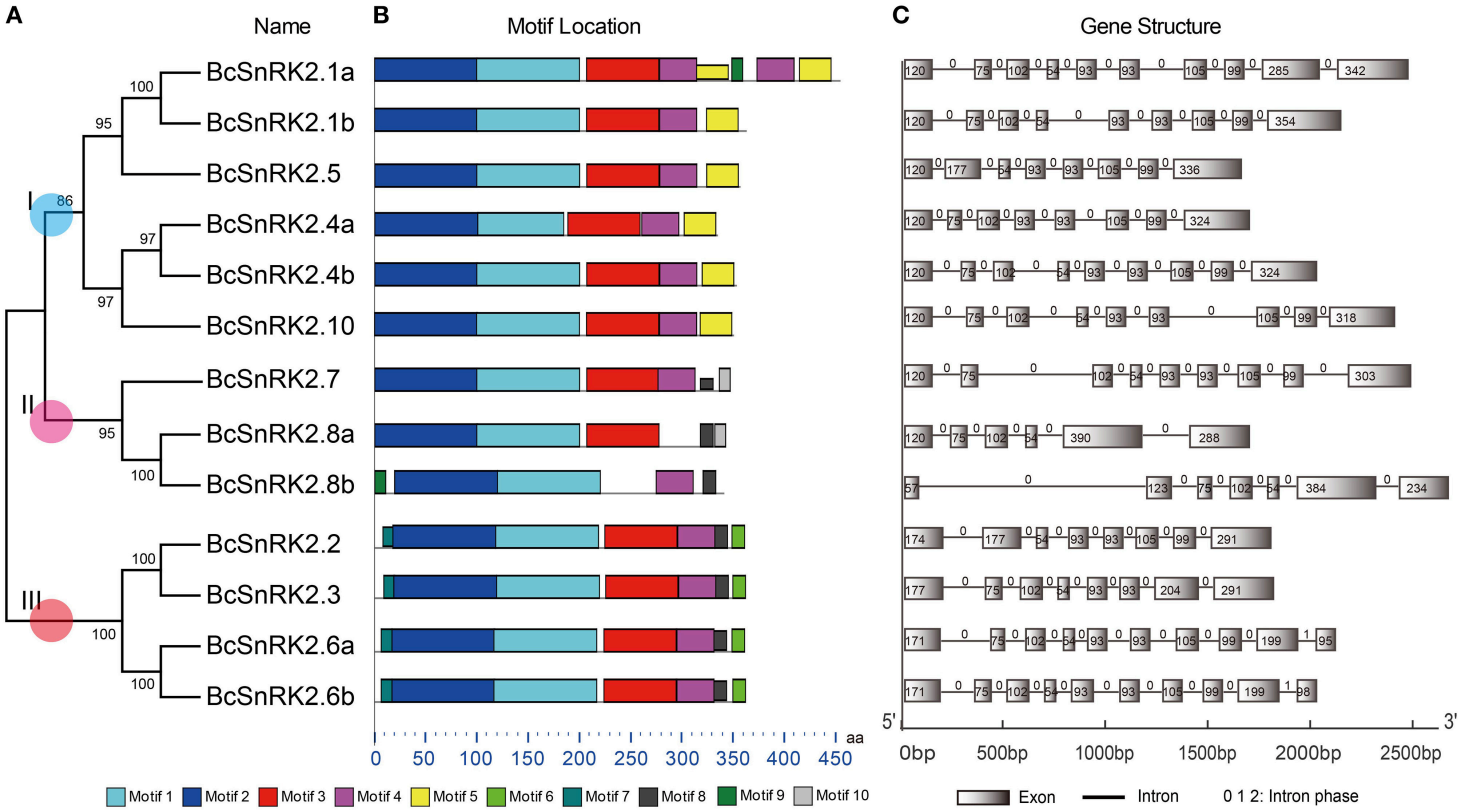
**Diagrammatic structure of BcSnRK2 genes. (A)** The unrooted phylogenetic tree resulting from the full-length amino acid alignment of all of the BcSnRK2 proteins in Pak-choi. The tree was constructed using maximum likelihood (ML) and bootstrap values calculated with 1000 replications using MEGA5 (Tamura et al., 2011). **(B)** The distribution of the conserved motifs in the SnRK2 genes from Pak-choi identified using MEME tool. The length of bars indicate the length of BcSnRK2 proteins. **(C)** Exons and introns of the BcSnRK2 genes. Each motif is represented by a number in the colored box. Introns and exons are represented by black lines and boxes, respectively. The numbers above the introns indicate intron phase. The numbers in the exons indicate the length of the exons. The length of bars indicate the length of *BcSnRK2* genes.

Due to the significant role of *SnRK*s in plants, investigation of their characteristics and the evolution of their function will support the breeding work in *B. rapa*, especially Pak-choi. To obtain *SnRK2* orthologs in Pak-choi, we designed primers according to the best blast hits from Chinese cabbage genome for RT-PCR amplification from Pak-choi DNA and cDNA libraries. We obtained 13 full-length *BcSnRK2* genes sequences by DNA sequencing (Supplementary Table 7), and the 13 deduced BcSnRK2 proteins all contained protein kinase domains, as detected using the SMART tool. Then, these genes were all clustered into the SnRK2 group with AthSnRK2s and BraSnRK2s in the phylogenetic tree, which was performed for all of the AthSnRKs, BraSnRK2s and BcSnRK2s (Supplementary Figure 4), confirming their accuracy. According to the homology with *AthSnRK2*s, the *BcSnRK2* genes were named as *BcSnRK2.1a, BcSnRK2.1b, BcSnRK2.2, BcSnRK2.3, BcSnRK2.4a, BcSnRK2.4b, BcSnRK2.5, BcSnRK2.6a, BcSnRK2.6b, BcSnRK2.7, BcSnRK2.8a, BcSnRK2.8b*, and *BcSnRK2.10* (Table 1, Figure 4A).

**Table 1:** ***BcSnRK2* genes in Pak-choi (*Brassica rapa* ssp. *chinensis*)**.

***Gene name***	**Acc ID**	**DNA(bp)**	**CDS(bp)**	**Intron**	**Size(aa)**	**Mass(Kda)**	**IP**	**Subcellular localization**
*BcSnRK2.1a*	KT236157	2441	1368	9	455	51.93	5.43	Nuclear
*BcSnRK2.1b*	KT236158	2113	1095	8	364	41.71	6.04	Nuclear
*BcSnRK2.2*	KT236159	1780	1086	7	361	41.00	4.46	Nuclear
*BcSnRK2.3*	KT236160	1786	1089	7	362	41.07	4.55	Nuclear
*BcSnRK2.4a*	KT236161	1678	1011	7	336	38.00	5.24	Cytoplasmic
*BcSnRK2.4b*	KT236162	2004	1065	8	354	40.39	6.20	Cytoplasmic
*BcSnRK2.5*	KT236163	1636	1077	7	358	41.16	5.09	Nuclear
*BcSnRK2.6a*	KT236164	2095	1086	9	361	40.88	4.69	Nuclear-Cytoplasmic
*BcSnRK2.6b*	KT236165	2000	1089	9	362	40.91	4.59	Nuclear
*BcSnRK2.7*	KT236166	2465	1044	8	347	39.39	4.64	Cytoplasmic
*BcSnRK2.8a*	KT236167	1670	1029	5	342	38.15	5.44	Nuclear-Cytoplasmic
*BcSnRK2.8b*	KT236168	2640	1029	6	342	38.07	5.62	Nuclear-Cytoplasmic
*BcSnRK2.10*	KT236169	2384	1059	8	352	40.22	6.36	Cytoplasmic

bp, base pair; aa, amino acids; CDS, coding sequence; pI, Isoelectric point.

Then, the sequences features of 13 *BcSnRK2* genes were specifically analyzed (Table 1). The length of their coding sequences ranged from 1000 to 1100 bps with the exception of *SnRK2.1a*, which contained 1368 bps. According to the number of their introns (Figure 4C), *BcSnRK2* genes could be classified into two types (Figure 1 and Table 1). The first type had 7–9 introns contained 11 genes. The second type included 2 genes that had 5 to 6 introns. Compared with *A. thaliana*, where most of the AthSnRK2s had 8 introns, except for *AthSnRK2.6* (9 introns) and *AthSnRK2.8* (5 introns), the structures of *BcSnRK2* genes were little changed. Moreover, in both *A. thaliana* and *Zea mays*, the lengths of the second to eighth exons respectively were 75, 102, 54, 93, 93, 105, and 99 (bps). Notably, the lengths of the second to eighth exons were 75, 102, 54, 93, 93, 105, and 99 (bps) in the *BcSnRK2* genes, which had 8 and 9 introns, with the exception of *BcSnRK2.5* (Figure 4C). However, the second exon of *BcSnRK2.5* was 177 bps which was equal to the sum of 102 and 55 bps.

### Protein properties and conservative analysis of SnRK2 in pak-choi

SnRK2 is a plant-specific Ser/Thr protein kinase family. To further understand the roles of this protein family, we analyzed the molecular properties and sequence characteristics of putative BcSnRK2 proteins. Among the 13 putative BcSnRK2 proteins, the size ranged from 336 to 455 aa, the isoelectric point ranged from 4.55 to 6.36, and the molecular weight ranged from 38.00 to 51.93 Kda (Table 1).

Based on previous reports, all SnRK2s have an N-terminal conserved catalytic domain similar to SNF1/AMP kinases and a short C-terminal regulatory domain that is not highly conserved. By using the MEME motif research tool, we identified 10 motifs in Pak-choi SnRK2 proteins (Figure 4B). Motif 1 and motif 2 were contained in the N-terminal of all genes, suggesting they belonged the protein kinase domain. In addition, motif 3 and motif 4 were also found in all genes, except for *SnRK2.8a* and *SnRK2.8b*. However, the motifs of the C-terminal were more similar within groups than between groups. Both motif 8 and motif 9 were present in group III, suggesting they were needed for the ABA response (Kulik et al., 2011).

**Figure 5:**
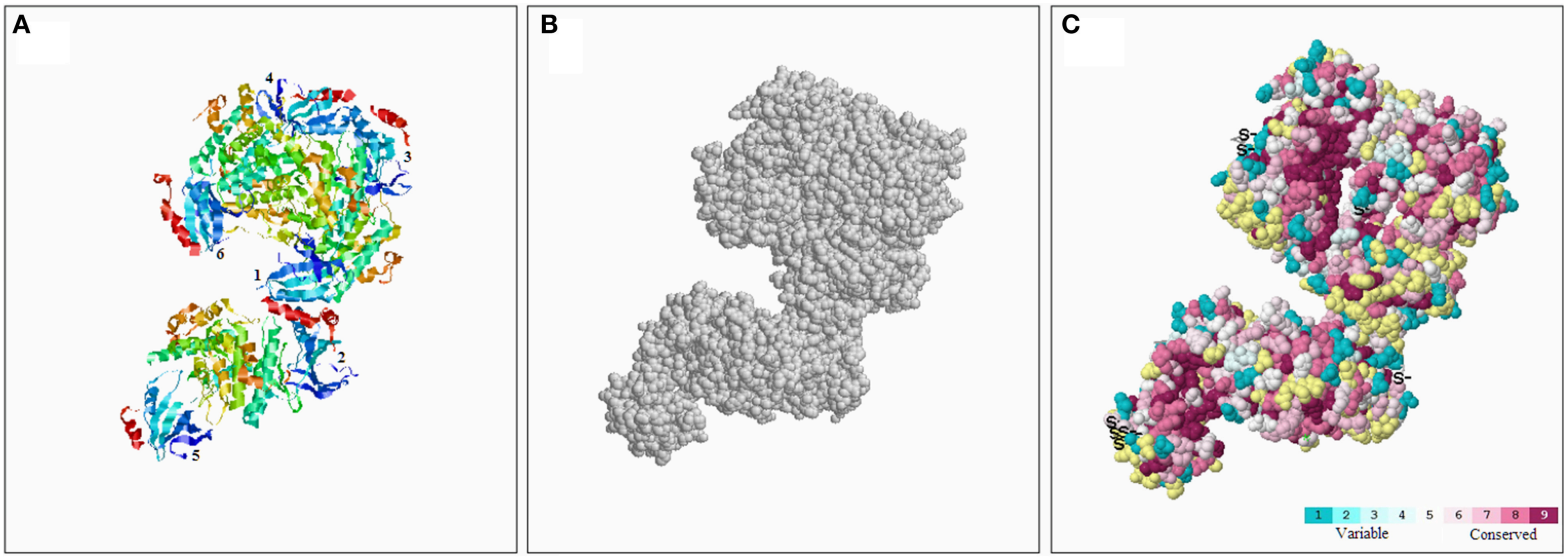
**Conserved evolution of SnRK2.6b—3D. (A)** Crystal structure of the putative SnRK2 protein based on SnRK2.6b. The six subunits are marked with 1 and 6, respectively. **(B)** The amino acids were presented as gray balls on the spatial structure. **(C)** Residues are colored according to their levels of conservation. The score is 1–9 as blue to purple, which represents viable to conserve.

To better understand the conserved amino acid distributions and the conserved spatial structural evolution of SnRK2 proteins in Pak-choi, we establish a three delimitation structural model of the BcSnRK2 protein (Figure 5) using the ConSurf web server on the basis of the structure of *AthSnRK2.6* and alignments of *A. taliana* and Pak-choi SnRK2 proteins (Supplementary Figure 5). The structural models of six subunits were also constructed (Supplementary Figure 6). The amino acids were represented by color balls; the deep red parts of the native protein has a high conservation score of 9, in contrast to the blue parts that were conserved with a low negative normalized score of 1 (Figure 5). In the putative structural model of the BcSnRK2 protein, the conserved region located in the inner part, and most residues of BcSnRK2 proteins were indicated on the cover.

Overall, the results of the protein structure and alignment analyses demonstrated the high evolutionary conservation of the SnRK2 gene family, especially between Pak-choi and *A. thaliana*.

### Subcellular localization of SnRK2.6a

**Figure 6:**
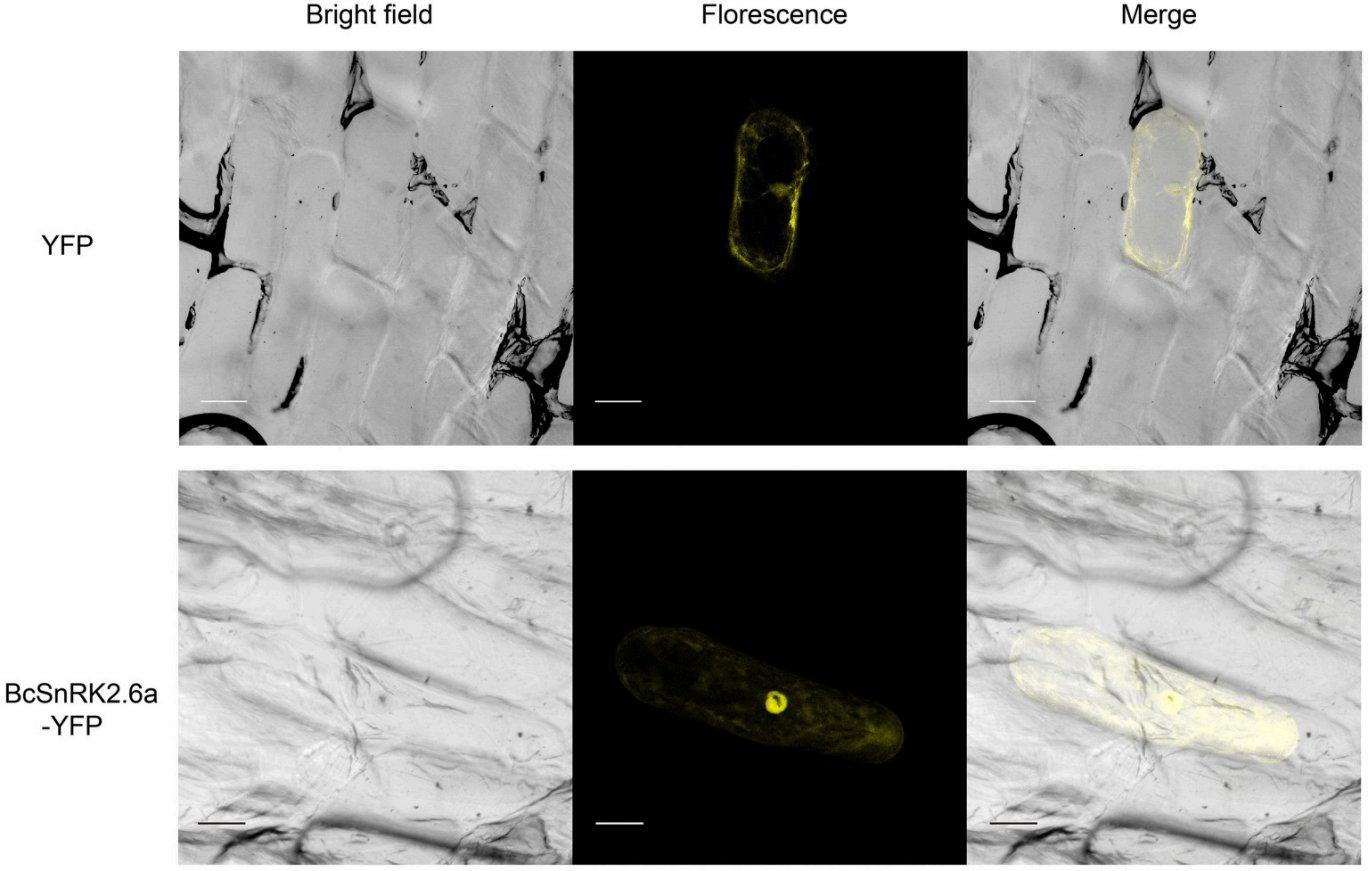
**Subcellular localization analysis of SnRK2.6a**. The subcellular localization analysis of the putative BcSnRK2.6a protein. The upper panel, the corresponding bright field, fluorescence, merged fluorescence image of YFP control; the lower panel, the corresponding bright field, fluorescence, merged fluorescence image of BcS2.6a-YFP. Scale bars: 20 μm.

According subcellular localization predictions, most of the putative *BcSnRK2* proteins were located in the nucleus. *BcSnRK 2.6a, BcSnRK 2.8a*, and *BcSnRK 2.8b* were located both in the nucleus and the cytoplasm, while *BcSnRK 2.4a, BcSnRK 2.4b, BcSnRK 2.7*, and *BcSnRK 2.10* were cytoplasmic (Table 1). To further test the subcellular localization of BcSnRK proteins, we used a transient expression system in onion epidermal cells. The yellow fluorescent marker protein (YFP) as a *BcSnRK 2.6a* tag can provide insights into its *in vivo* (dynamic) subcellular localization. When YFP alone was expressed, fluorescence was observed in the cytosol and nucleus (Figure 6, upper panel), while the yellow fluorescence of the *BcSnRK 2.6a* fusion proteins were also both nuclear and cytoplasmic, but the nuclear expression was more obvious (Figure 6, lower panel). Thus, the empirical results for the subcellular localization agreed with the prediction results for the subcellular localization.

### Expression of BcSnRK2 genes under low temperature and ABA treatments

**Figure 7:**
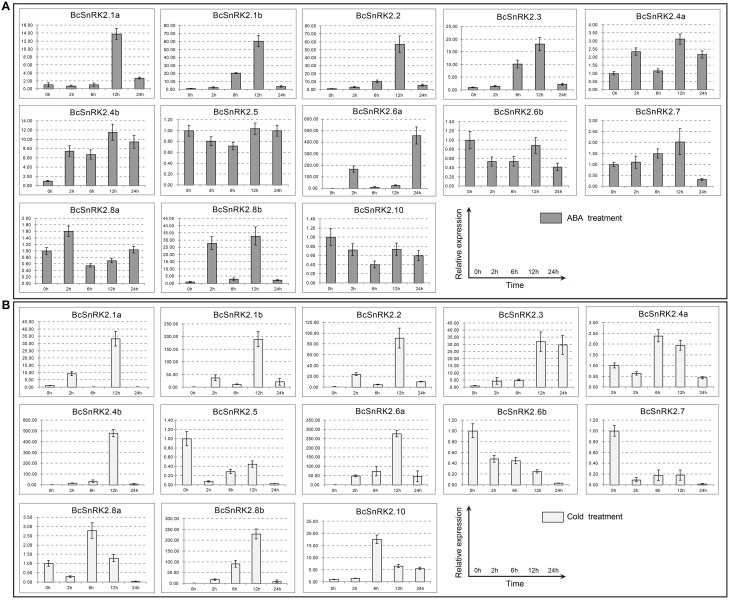
**Expression patterns of the 13 BcSnRK2s under ABA and cold treatments**. Pak-choi plants were subjected to ABA **(A)** and cold treatments **(B)**. The 13 stress-inducible BcSnRK2s were differently expressed in the leaves in response to abiotic stressors and their transcript levels were quantified against BcGAPDH transcript levels using 2^−ΔΔCT^, ΔΔCT = ΔCT (treated sample)−ΔCT (untreated sample), ΔCT = CT_target_-CT_BcGAPDH_.

The results of the gene structure and phylogenetic analysis suggested that BcSnRK2s were strictly conserved with AthSnRK2s. To identify the function of BcSnRK2s in response to abiotic stresses, we analyzed the expression of *BcSnRK2* genes using real-time PCR under low temperature and ABA treatments (Figure 7).

The induced expression patterns of all group III members were higher than those for group I, except for *BcSnRK2.6b*, which was reduced significantly. Additionally, *BcSnRK2.6a* not only had the highest expression but also had two peak values at 2 h and 24 h. Members of subgroup I, except for *BcSnRK2.10*, were reduced slightly, *BcSnRK2.5* remained stable, and *BcSnRK2.1a, BcSnRK2.1b, BcSnRK2.4a, and BcSnRK2.4b* were induced by the ABA treatment. Group II members, *BcSnRK2.7* and *BcSnRK2.8b* were induced and *BcSnRK2.8a* showed almost no change. Overall, group III expression was consistent with the idea that its members could be activated by ABA, and function in ABA signal transduction by phosphorylating ABFs (Fujii et al., 2007; Kulik et al., 2011; Wang et al., 2013). However, most of the *SnRK2* genes, especially *SnRK2.1b and SnRK2.8b*, could also be activated by ABA treatment.

Under a low temperature, the expression of group I members were induced, except for *BcSnRK2.5. BcSnRK2.5*, which were reduced strongly at 2 h, and then slowly rebounded at 6 to 12 h, then reduced strongly again at 24 h. In group III, with the exception of *BcSnRK2.6b*, which was reduced by a low temperature, the rest of members were all induced sharply, especially *BcSnRK2.6a*. The expression of *BcSnRK2.7* and *BcSnRK2.8b* in group II changed strongly. However, *BcSnRK2.7* was reduced and *BcSnRK2.8b* was induced. These results clearly reveal distinct and common expression patterns of *BcSnRK2* genes in response to ABA and low temperature treatments, and some *BcSnRK2* genes either co-responded or specifically responded to ABA and cold stress, such as *BcSnRK2.6a* which was induced strongly by both stimuli.

**Figure 8:**
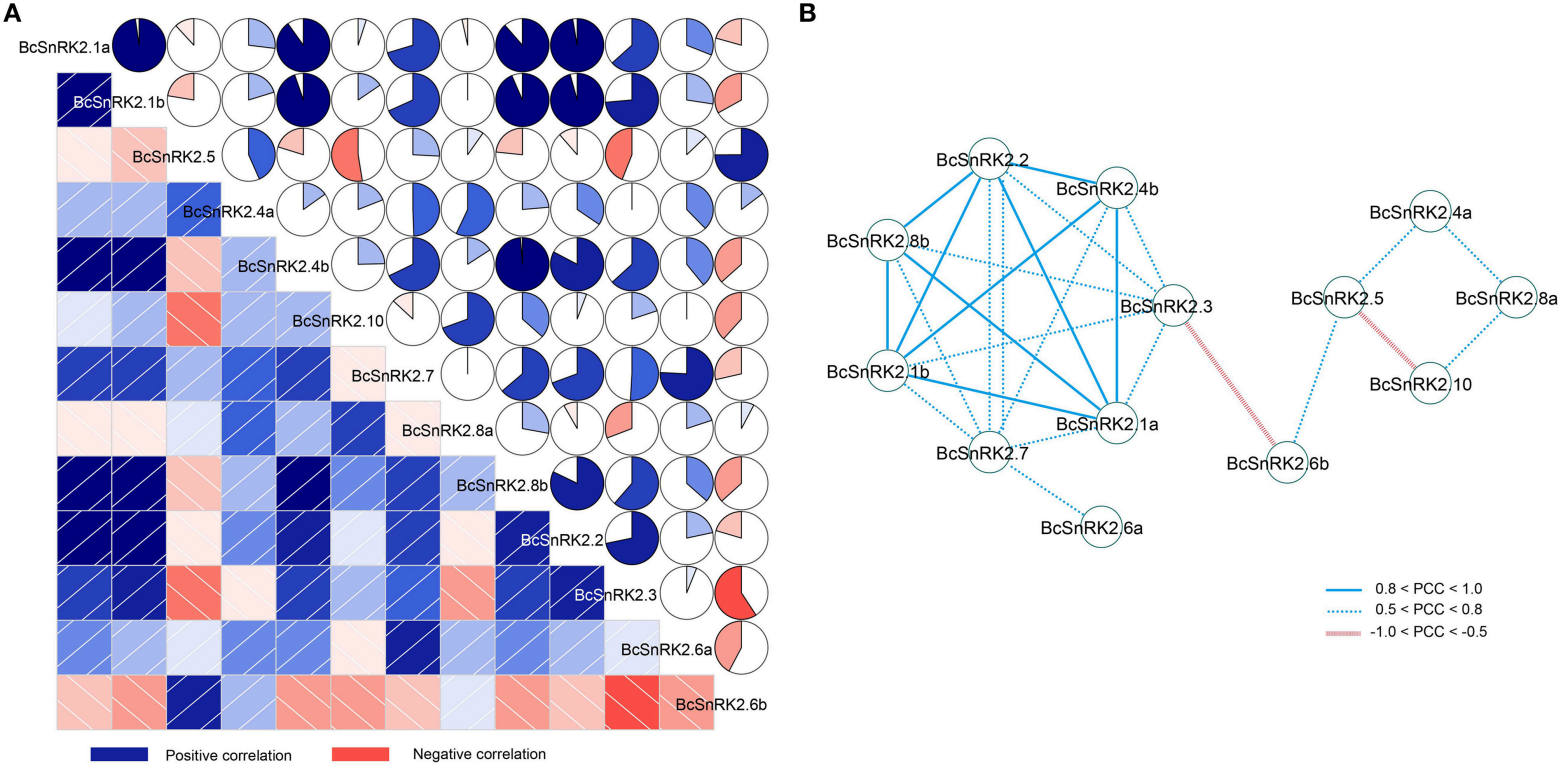
**Correlations and co-regulatory networks of 13 BcSnRK2 under cold and ABA treatments**. **(A)** Correlation analysis by using the R package program. Lower squares: correlations indicated by color and intensity of shading; upper: circular symbols. Each correlation is shown by the shades of blue and red and the size of the fan shape. Blue and red indicate a positive correlation and negative correlation, respectively. **(B)** Co-regulatory networks. The co-regulatory networks of 13 BcSnRK2 under cold and ABA treatments were established based on the Pearson correlation coefficients (PCC) of these gene pairs using transformed qPCR data, which involved 13 nodes and 25 regulatory edges. The PCC of co-regulatory gene pairs was considered significant at the 0.05 significance level (*p*-value), and different edge line styles indicate the different significance levels of the co-regulated gene pairs.

In addition, there were four pairs of duplicated *SnRK2* genes in Pak-choi, *BcSnRK2.1a* and *BcSnRK2.1b, BcSnRK2.4a* and *BcSnRK2.4b, BcSnRK2.6a* and *BcSnRK2.6b*, and *BcSnRK2.8a* and *BcSnRK2.8b*, and their expression patterns were differentiated. Under ABA and low temperature treatments, *BcSnRK2.6a* was up-regulated and *BcSnRK2.6b* was down-regulated; *BcSnRK2.1b, BcSnRK2.4b*, and *BcSnRK2.8b* were induced more than *BcSnRK2.1a, BcSnRK2.4a*, and *BcSnRK2.8a.* To investigate the connection between these genes, correlation and co-regulatory networks were established based on the PCCs of the relative expression of the genes (Figure 8). There were close correlations between some genes, such as *BcSnRK2.1a* and *BcSnRK2.1b* and *BcSnRK2.4b* and *BcSnRK2.8b.* Additionally, there were inverse correlations between *BcSnRK2.6b* and almost all of other *BcSnRK2s*, except *BcSnRK2.5* and *BcSnRK2.4a* (Figure 8A). All PCCs that were significant at the 0.05 significance level (*p*-value) and with a correlation of more than 0.5 were collected and visualized in the Cytoscape program to construct ABA- and low temperature-related co-regulatory networks of SnRK2 genes (Figure 8B). In the co-regulatory networks, there were 13 nodes and 27 edges. While 2 pairs (*BcSnRK2.3* and *BcSnRK2.6b* and *BcSnRK2.5* and *BcSnRK2.10)* showed negative correlations, the rest of the co-regulatory gene pairs had positive significant correlations. Additionally, with the exception of *SnRK2.6a* and *SnRK2.6b*, the members of group III had a close relationship with other groups. However, there was no correlation observed between most of the duplicated genes. The networks could reflect the divergence trend of the duplicated genes. The expansion of the gene family depicted in the network, could help plants adapt to the changing environment by increasing cooperation and obtaining new functions.

## Discussion

During evolution, genome duplication not only provided abundant genetic materials for evolution but might also have produced bulk genetic variations, supporting plants to adapt better to diversified environments, such as drought, high salinity, and extreme temperatures. *SnRK2* genes, which are a significant part of the ABA signal pathway, are involved in many processes that help plants resistant environmental pressures. In previous reports, *SnRK2* genes were identified in Algal and were demonstrated to have originated in the last common ancestor of land plants (Wang et al., 2015). We identified 2 *SnRK2* genes in *C. reinhardtii*, consistent with the previous reports. In previous reports (Hauser et al., 2011), *SnRK2* genes could be divided into three group and group III is most ancient group. However, in *Malus prunifolia* (Shao et al., 2014), SnRK2s clustered into four groups. With the help of a phylogenetic analysis using *B. rapa, A. thaliana* and other seven plant species, including 3 eudicots (*C. papaya, P. trichocarpa* and *V. vinifera*), 1 basal angiosperm (*A. trichopoda*), 1 lycophyte (*S. moellendorffii*), 1 physcomitrella (*P. patens*), and 1 chlorophyta (*C. reinhardtii*), we divided SnRK2s into three groups. Additionally, based on the footprint and previous finding that group III was the most ancient group and group I was the most recent group (Umezawa et al., 2010), we inferred that both group I and group II formed from group III. In addition, the expansion of group I played a major role in the expansion of the SnRK2 gene family.

WGDs have been proposed to be important in the evolution of complexity in multicellular eukaryotes (Edger and Pires, 2009). During the different WGD events, genes experienced duplication, differentiation and lost. Compared with *A. thaliana*, except for α, β, γ WGD events, Brassicas crops experienced an additional WGT event that happened approximately 13–17 MYA (Wang et al., 2011; Cheng et al., 2013). The gene balance hypothesis predicts that genes whose products participate in macromolecular complexes or in transcriptional or signaling networks are more likely to be retained, thus avoiding network instability caused by the loss of one member (Birchler and Veitia, 2007; Lou et al., 2012). Consistent with this hypothesis, genes that encode transcription factors and signal transduction components exhibit preferential retention in *A. thaliana* after WGD events (Maere et al., 2005; Freeling and Thomas, 2006; Bekaert et al., 2011). Similarly, in our study, all *SnRK2*s were retained in *B. rapa* and *B. oleracea* after a WGT event that happened after the split with *A. thaliana* from the most recent common ancestor. In addition, retention of the *BraSnRK2* genes were more preferred than neighboring genes. The data that we obtained is consistent with the gene balance hypothesis and demonstrates that *SnRK2*s play key roles in the adaptation of Brassica crops.

In *Arabidopsis*, sorghum and rice, 10 SnRK genes have been well-annotated (Kulik et al., 2011). Here we identified, 15 *BraSnRK2s*, 6 *CpaSnRK2s*, 14 *PtrSnRK2s*, 8 *VviSnRK2s*, 4 *AtrSnRK2s*, 4 *PpaSnRK2s*, 3 *SmoSnRK2s*, and 2 *CreSnRK2s*. Based on previous results that most of the *SnRK2s* responded to environmental stress, we cloned 13 *BcSnRK2* genes in stress-induced Pak-choi DNA and cDNA libraries, which including ABA, salinity, cold, heat, and osmotic treatments. All of the cloned genes corresponded to *AthSnRK2* genes, except for *AthSnRK2.9* (Figure 4). Based on the footprint of *SnRK2* genes, *SnRK2.9*, which belonged to group I, were found after the α event in *A. thaliana, B. rapa*, and *B. oleracea.* Given our approach, we could not confirm whether *SnRK2.9* was lost in Pak-choi or the sequence changed so much that we could not obtained it by cloning.

SnRK2 is a plant-specific Ser/Thr protein kinase family. In our study, we found that there are lots of common characteristics in their structures, such as the number and the lengths of exons among the SnRK2 genes in *A. thaliana*, Pak-choi and even maize. Previous studies reported the conserved gene structure of the SnRK2 gene formed from moss (Huai et al., 2008). Based on the multiple sequence alignment of SnRK2s, we establish a three delimitation structure model of BcSnRK2s (Supplementary Figure 4 and Figure 6). All results showed that BcSnRK2s were strictly conserved with *A. thaliana* at the N-terminus. Above all, *SnRK2* genes changed little during evolution, and they might have common features playing important roles in plant growth and development.

Results presented by several laboratories provide evidence that members of the *SnRK2* gene family could be separated into ABA-dependent and ABA-independent groups. In *A. thaliana*, group III (*SnRK2.2, SnRK2.3, and SnRK2.6*) could be active by ABA treatment, and others could not. Additionally, the regulation of ABA signal transduction occurs through protein–protein interactions between PYLs and PP2Cs, and also between PP2Cs and SnRK2s (Fujii and Zhu, 2009; Umezawa et al., 2010). However, the expression of SnRK2s is different in different plants. In maize, expression of *SnRK2* genes were enhanced in response to ABA (*ZmSnRK2.2, ZmSnRK2.4, ZmSnRK2.5, ZmSnRK2.7, and ZmSnRK2.10*), and cold (*ZmSnRK2.3, ZmSnRK2.7*). The genes that respond to ABA do not all belong to subgroup III. In our results, besides subgroup III, the genes of subgroup II were also induced by ABA. In 2004, according to Boudsocq's report, all of the *AthSnRK2* genes did not respond to low temperature. With the establishment of the decuple (*snrk2.1/2/3/4/5/6/7/8/9/10*) and septuple (*snrk2.1/4/5/7/8/9/10*) SnRK2 mutants, the idea that SRK2D/SnRK2.2, SRK2E/SnRK2.6, and SRK2I/SnRK2.3 are essential components of the osmotic stress response and ABA signaling have strengthened (Fujii et al., 2011). In addition, a structural analysis of a SnRK2-PP2C complex showed that PP2C recognition by SnRK2s and by PYR/PYL/RCAR ABA receptors is quite similar. Moreover, SnRK2.6/SRK2E interacts with PP2Cs through not only the kinase domain but also through its C-terminal ABA box (which nearly corresponds to domain II), whose binding to PP2Cs is not affected by ABA or its receptors (Soon et al., 2012). Our results showed that most of the *BcSnRK2* genes could be activated by cold and *BcSnRK2.6a* could be induced strongly both by ABA and cold. Thus, our result support that SnRK2s are global regulators that function at the intersection of multiple signaling pathways, and in ABA-dependent and -independent signaling pathways.

After duplication, genes might become non-functional (pseudogenized or silenced, also called gene death), neofunctional (a novel function), or subfunctional (dividing the original function) (Innan and Kondrashov, 2010). There were four pairs of duplicated *BcSnRK2* genes. *BcSnRK2.6a* was induced and *BcSnRK2.6b* was reduced by ABA suggesting that they diverged from their original function. On the other hand, both of *BcSnRK2.4a* and *BcSnRK2.4b* were barely activated by ABA, and *BcSnRK2.4b* were induced rapidly by low temperature. Similar expression patterns also found for *BcSnRK2.1a, BcSnRK2.1b, BcSnRK2.8a*, and *BcSnRK2.8b*. Above all, the functions of *SnRK2s* diverged following the change in the environment.

In conclusion, we performed a genome-wide analysis of the SnRK2 gene family to investigate its evolutionary pattern and footprint. We also isolated and characterized 13 *BcSnRK2* genes from Pak-choi and elucidated their structure, conserved motifs and expression patterns under low temperature and ABA treatments to further understand this gene family. We demonstrated that these genes were preferential retained and that show *SnRK2s* co-respond to cold stress and ABA signal transduction in Pak-choi. Our results help to further the understanding of the function and molecular mechanisms of *SnRKs* during the plant stress response and support the breeding of Pak-choi.

## Author contributions

Conceived and designed the experiments: ZH, JT, XH, and WD. Performed the experiments: ZH, JT, ZW and WD. Analyzed the data: JT, ZH, WD, and XS. Contributed reagents/materials/analysis tools: XH, JT, XS, and WD. Wrote the paper: ZH, WD, and JT.

### Conflict of interest statement

The authors declare that the research was conducted in the absence of any commercial or financial relationships that could be construed as a potential conflict of interest.
