# Supplementary material for: Molecular evolution, characterization, and expression analysis of SnRK2 gene family in Pak-choi (Brassica rapa ssp. chinensis)
Source: Front Plant Sci. 2015 Oct 21;6:879. doi: 10.3389/fpls.2015.00879 (PMC4617174; doi:10.3389/fpls.2015.00879)
Supplement: Supplementary file 1 [file DataSheet1.PDF]

Supplementary Material

Molecular evolution, characterization and expression analysis of SnRK2 gene family in Pak-choi (*Brassica rapa* ssp. *chinensis*)

Zhinan Huang[1], Jun Tang[1,2], Weike Duan[1], Zhen Wang[1], Xiaoming Song[1], Xilin Hou[1]*

Correspondence: Prof: Xilin Hou: hxl@njau.edu.cn

Supplementary Figure 1-6

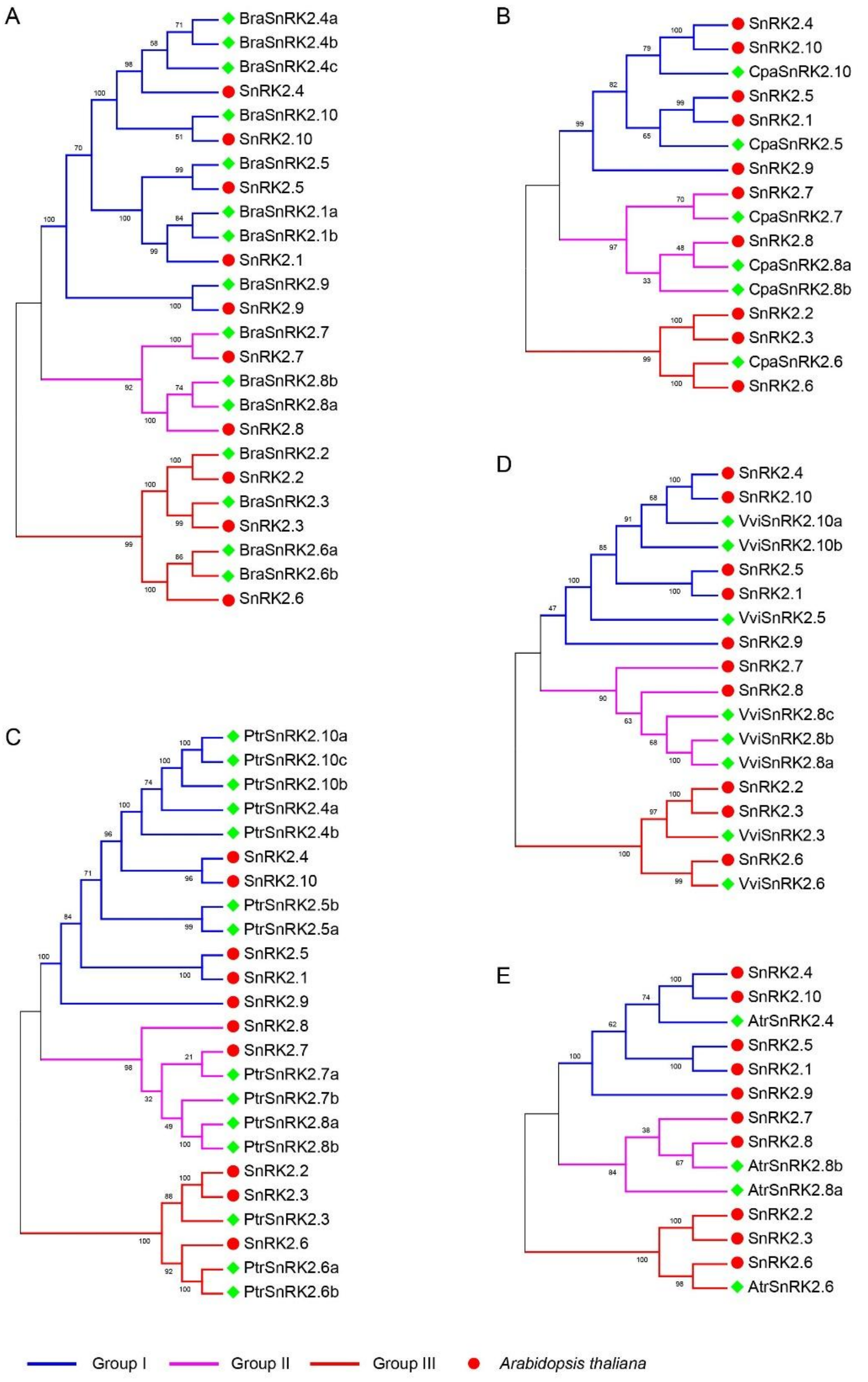

Supplementary Figure 1. Phylogenetic trees of SnRK2 genes in *A. thaliana* (Ath) and *B.rapa (Bra)* (A), *C. papaya* (Cpa) (B), *P. trichocarpa* (Ptr) (C), *V. vinifera* (Vvi) (D), *A. trichopoda* (Atr) (E), respectively. Group I, II and III are shaded blue, pink and red. Red balls indicate AthSnRK2s. Green squares indicate SnRK2s in other species.

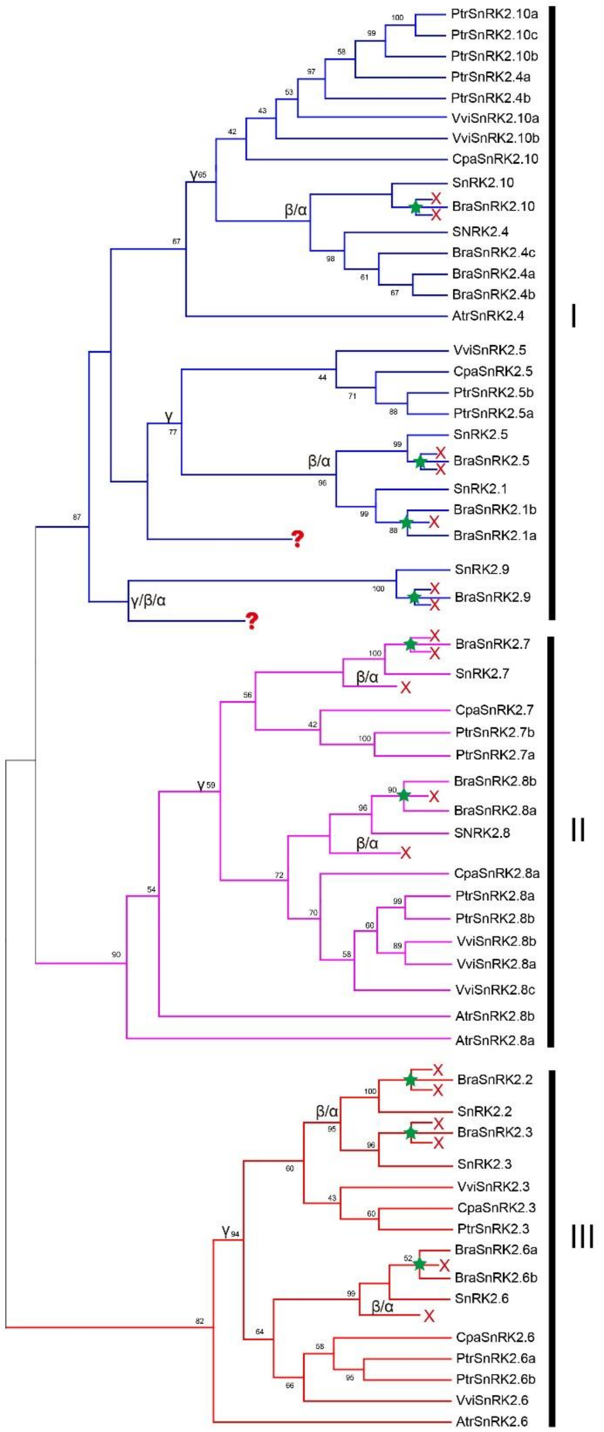

Supplementary Figure 2. Phylogenetic analysis of the SnRK2 gene family in angiosperms. Protein sequences were aligned and a phylogenetic tree was constructed by maximum likelihood (ML) using MEGA5 (Tamura et al., 2011). Gene losses are indicated by X. α, β, and γ duplications as well as the Brassica triplication are indicated. Green stars indicate the Brassica triplication. Group I, II and III are shaded blue, pink and red. *A. thaliana* (Ath), *B. rapa* (Bra), *C. papaya* (Cpa), *P. trichocarpa* (Ptr) and *V. vinifera* (Vvi)

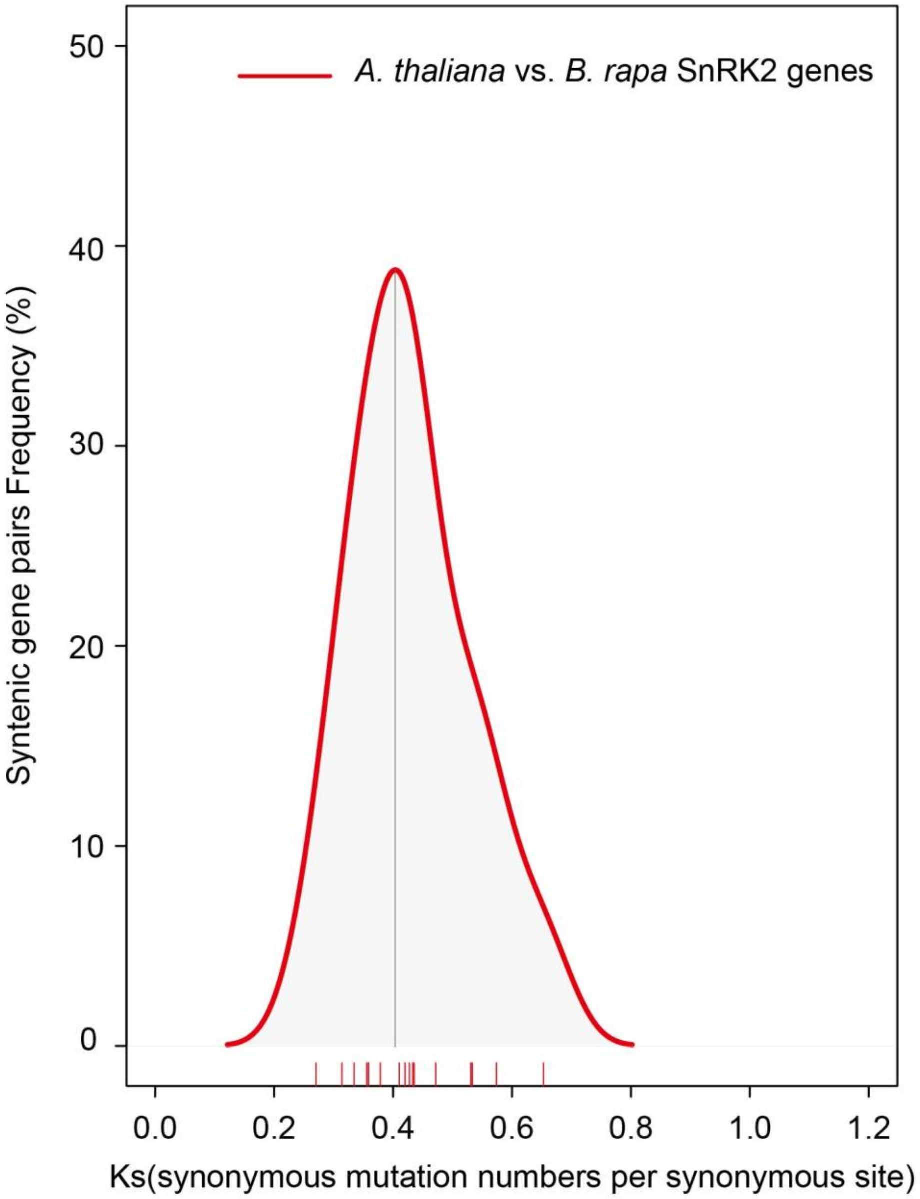

Supplementary Figure 3. Pairwise comparison of K_s values for SnRK2 homologous genes in *Brassica rapa* and *Arabidopsis thaliana*.

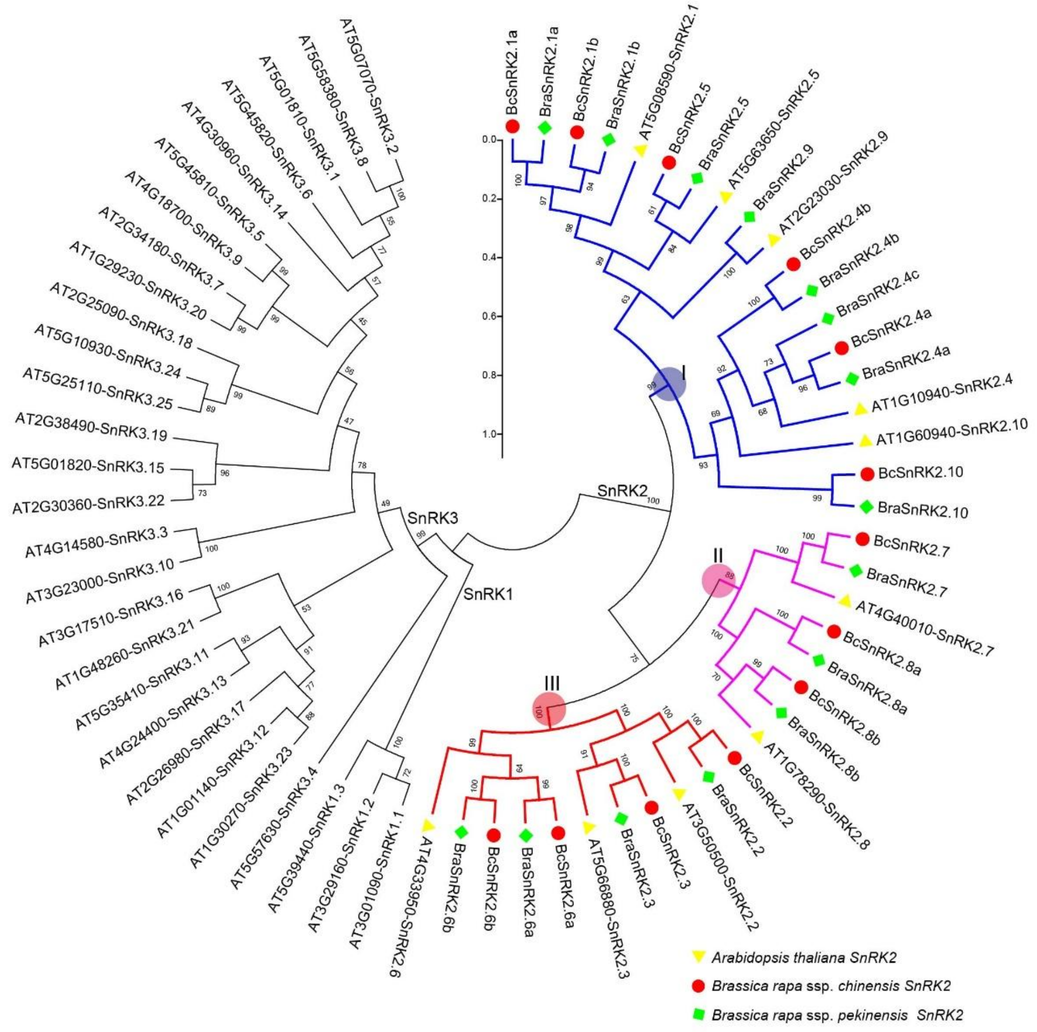

Supplementary Figure 4. Phylogeny of 13 BcSnRK2s, 15 BraSnRK2s and 38 AthSnRKs. The amino acid sequences of 13 SnRK2s in Pak-choi, 15 SnRK2s in *B. rapa* and 38 SnRKs in *A. thaliana* were aligned and used to construct a phylogenetic tree using maximum likelihood (ML) and bootstrap values were calculated with 1000 replications using MEGA5 (Tamura et al., 2011). Group I, II and III are shaded blue, pink and red. The yellow triangles indicate *A. thaliana*, the green squares indicate *B. rapa* and the red balls indicate Pak-choi.

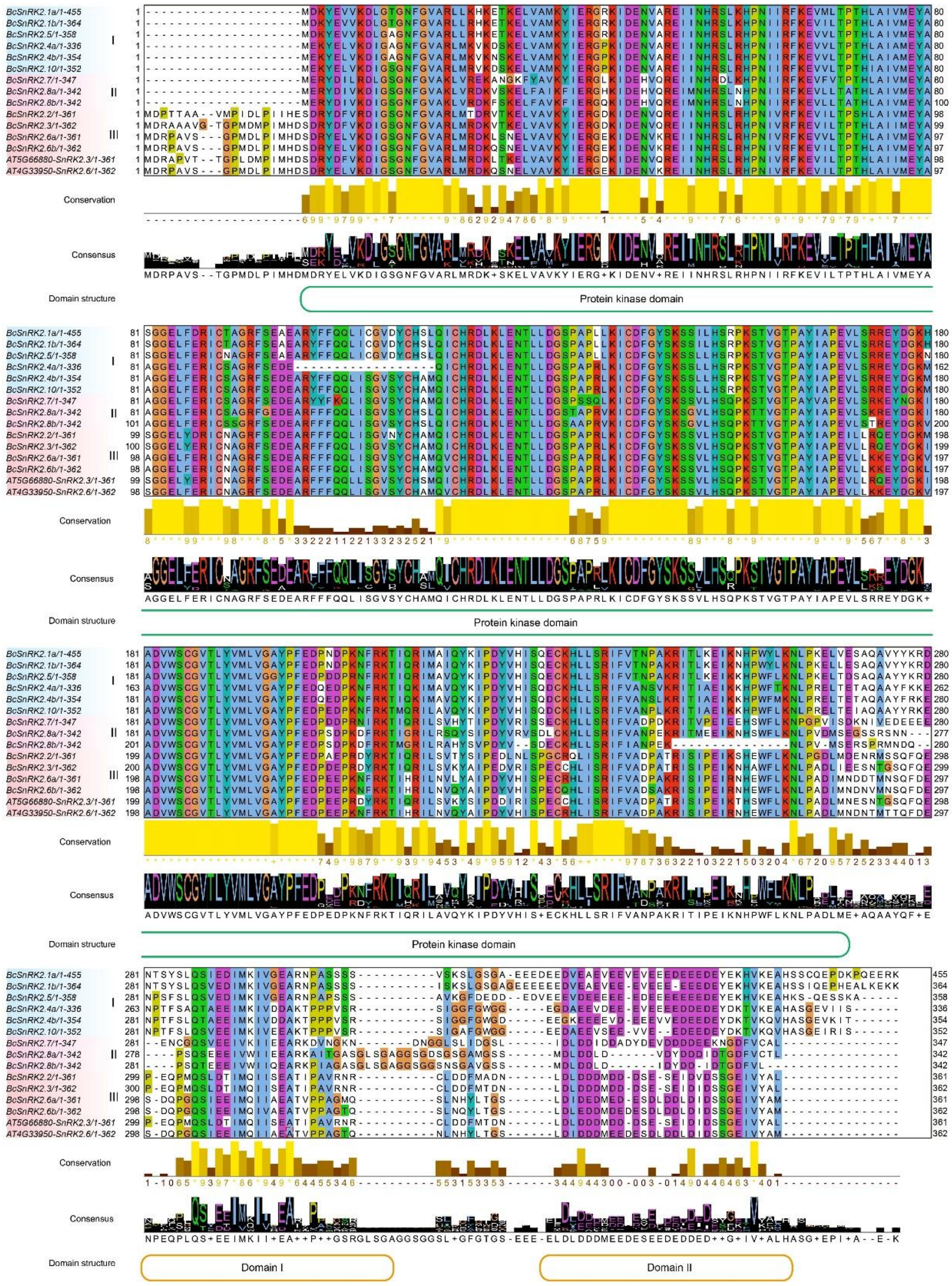

Supplementary Figure 5. Alignments of *A. thaliana* and Pak-choi SnRK2 proteins. Conservations and consensus were demonstrated under the alignments. Characteristic domains were marked: activation loop (green box), domain I and domain II (orange boxed).

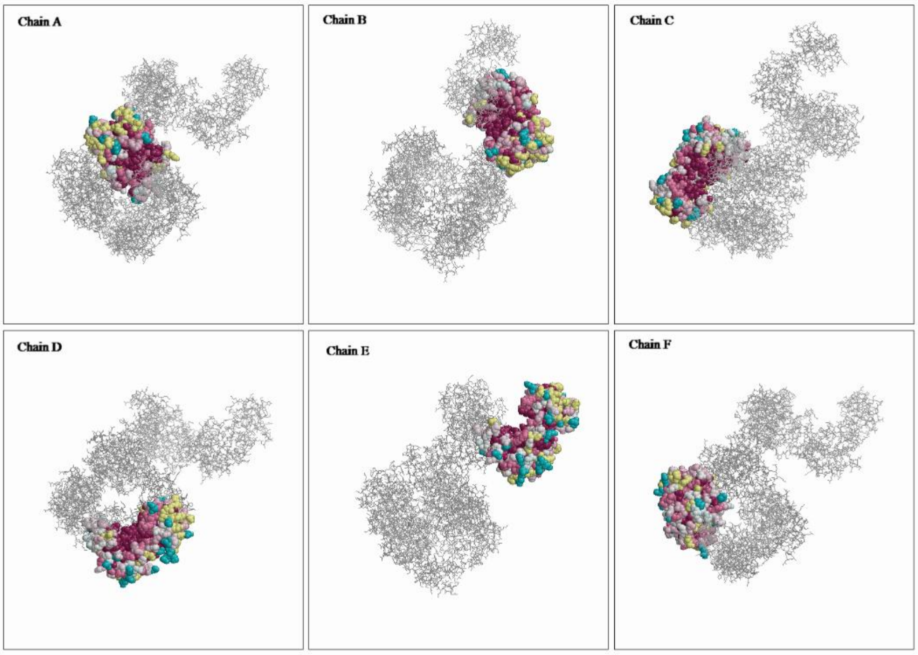

Supplementary Figure 6. Conservation level of six subunits. The score is 1 to 9 as blue to purple.
